# Financial Toxicity Among Patients With Breast Cancer Worldwide: A Systematic Review and Meta-analysis

**DOI:** 10.1001/jamanetworkopen.2022.55388

**Published:** 2023-02-08

**Authors:** Anam N. Ehsan, Catherine A. Wu, Alexandra Minasian, Tavneet Singh, Michelle Bass, Lydia Pace, Geoffrey C. Ibbotson, Nefti Bempong-Ahun, Andrea Pusic, John W. Scott, Rania A. Mekary, Kavitha Ranganathan

**Affiliations:** 1 Program in Global Surgery and Social Change, Harvard Medical School, Boston, Massachusetts; 2 Department of Surgery, Brigham and Women’s Hospital, Boston, Massachusetts; 3 Harvard Medical School, Boston, Massachusetts; 4 Department of Plastic Surgery, University of California, Orange; 5 School of Pharmacy, MCPHS University, Boston, Massachusetts; 6 Countway Library of Medicine, Harvard Medical School, Boston, Massachusetts; 7 United Nations Institute for Training and Research, Palais des Nations, Geneva, Switzerland; 8 The Global Surgery Foundation, Geneva, Switzerland; 9 Center for Healthcare Outcomes and Policy, Department of Surgery, University of Michigan, Ann Arbor

## Abstract

**Importance:**

Financial toxicity (FT) is the negative impact of cost of care on financial well-being. Patients with breast cancer are at risk for incurring high out-of-pocket costs given the long-term need for multidisciplinary care and expensive treatments.

**Objective:**

To quantify the FT rate of patients with breast cancer and identify particularly vulnerable patient populations nationally and internationally.

**Data Sources:**

A systematic review and meta-analysis were conducted. Four databases—Embase, PubMed, Global Index Medicus, and Global Health (EBSCO)—were queried from inception to February 2021. Data analysis was performed from March to December 2022.

**Study Selection:**

A comprehensive database search was performed for full-text, English-language articles reporting FT among patients with breast cancer. Two independent reviewers conducted study screening and selection; 462 articles underwent full-text review.

**Data Extraction and Synthesis:**

A standardized data extraction tool was developed and validated by 2 independent authors; study quality was also assessed. Variables assessed included race, income, insurance status, education status, employment, urban or rural status, and cancer stage and treatment. Pooled estimates of FT rates and their 95% CIs were obtained using the random-effects model.

**Main Outcomes and Measures:**

FT was the primary outcome and was evaluated using quantitative FT measures, including rate of patients experiencing FT, and qualitative FT measures, including patient-reported outcome measures or patient-reported severity and interviews. The rates of patients in high-income, middle-income, and low-income countries who incurred FT according to out-of-pocket cost, income, or patient-reported impact of expenditures during breast cancer diagnosis and treatment were reported as a meta-analysis.

**Results:**

Of the 11 086 articles retrieved, 34 were included in the study. Most studies were from high-income countries (24 studies), and the rest were from low- and middle-income countries (10 studies). The sample size of included studies ranged from 5 to 2445 people. There was significant heterogeneity in the definition of FT. FT rate was pooled from 18 articles. The pooled FT rate was 35.3% (95% CI, 27.3%-44.4%) in high-income countries and 78.8% (95% CI, 60.4%-90.0%) in low- and middle-income countries.

**Conclusions and Relevance:**

Substantial FT is associated with breast cancer treatment worldwide. Although the FT rate was higher in low- and middle-income countries, more than 30% of patients in high-income countries also incurred FT. Policies designed to offset the burden of direct medical and nonmedical costs are required to improve the financial health of vulnerable patients with breast cancer.

## Key Points

**Question:**

What is the rate of patients incurring financial toxicity as a result of breast cancer care nationally and internationally?

**Findings:**

In this systematic review and meta-analysis, the pooled rate of financial toxicity for patients with breast cancer was 78.8% in low- and middle-income countries and 35.3% in high-income countries.

**Meaning:**

These findings suggest that patients with breast cancer worldwide are at risk for financial toxicity; policies designed to offset the burden of direct medical costs, through expansion of health care coverage, and direct nonmedical as well as indirect costs, through interventions such as transportation and childcare facilities, are required to improve the financial health of vulnerable patients with breast cancer.

## Teaser

This systematic review and meta-analysis examines the rate of financial toxicity among patients with breast cancer worldwide.

## Introduction

Financial toxicity (FT) is the negative impact that the monetary burden of medical care can have on patients’ well-being, fiscal security, and overall health.^1^ Contributors to FT include direct medical, direct nonmedical, and indirect costs.^2^ Direct medical costs include hospitalizations, pharmaceutical bills, laboratory charges, and outpatient treatment–related expenses.^2^ Direct nonmedical and indirect costs include transportation, food and lodging, and expenses linked to disease-related employment loss.^2^ Nonmedical and indirect expenses are not generally covered by insurance and financial aid programs and contribute to out-of-pocket costs (OOPCs).^3^ Catastrophically high OOPCs are known to negatively impact medical care and psychosocial well-being.^4^

Compared with other chronic conditions, patients with cancer are at risk for higher OOPCs.^3,5,6^ Breast cancer care in particular may be associated with high FT given the need for screening and diagnosis, multidisciplinary care, and longitudinal follow-up; notably, gender also affects financial security.^4,7^ However, the financial burden of breast cancer care worldwide is not well-characterized. Most studies examining breast cancer–related FT focus on high-income countries (HICs), with little attention given to low- and middle-income countries (LMICs).^8,9^ Poverty is a major barrier to accessing care in LMICs, where patients often bear a substantial portion of the financial costs of treatment while also facing greater poverty. Patient financial concerns are associated with delayed breast cancer diagnoses and higher mortality.^10^ Establishing the global extent of FT and comparing the economic burden of disease in different populations is imperative to help policy makers prioritize funding of breast cancer care infrastructure.

To address this gap, we quantified the FT rate among patients with breast cancer in high-, middle-, and low-income countries through a meta-analysis. The comparison of FT across countries with different economic and health care landscapes may shed light on the efficacy of varying health care financing strategies and social safety net systems on FT. Developing this understanding is the first step to creating effective interventions that not only reduce financial stress on low-income patients but also increase access to care and improve breast cancer outcomes worldwide.

## Methods

### Overview

We conducted a systematic review and meta-analysis of studies exploring FT in individuals with breast cancer. This study did not require institutional review board approval because it is a review article using publicly available data sources. Informed consent was not needed because no patient data were used, in accordance with 45 CFR §46. A protocol for this review was developed using the Preferred Reporting Items for Systematic Reviews and Meta-analyses (PRISMA) reporting guideline and registered in PROSPERO (CRD42021228015).^11^

### Search Strategy and Selection Criteria

Original, full-text, English-language articles about FT related to breast cancer screening, diagnosis, and treatment were included. Records focusing on cost to the health system, rather than to individuals, or lacking stratified breast cancer data were excluded. Abstracts, editorials, commentary, reviews, and articles without full text were excluded.

Four databases—Embase (Elsevier), PubMed, Global Index Medicus, and Global Health (EBSCO)—were queried from inception to February 2021. A robust search strategy was developed by the medical librarian (M.B.) and the lead author (C.A.W.). Relevant articles for the concepts “breast cancer” and “out of pocket finances/financial stress” were retrieved (eAppendix in Supplement 1). All records were imported into Covidence for screening.

### Study Assessment and Data Extraction

A standardized data extraction tool was developed and validated by 2 authors (C.A.W. and A.M.) independently extracting a subset of records to ensure consistency and minimize bias. Deduplication was conducted manually and using Covidence algorithms. Two authors (C.A.W. and A.M.) independently performed title and abstract review of all records, followed by full-text review of remaining records. Conflicts were resolved by third author (K.R.). The primary outcome was rate of FT among patients with breast cancer.

### Study Categorization

Multiple definitions of FT were used across the studies. To synthesize heterogeneous data, quantitative and qualitative categories were established (Table 1). Quantitative methods included rate of patients experiencing FT (18 studies).^4,12,13,14,15,16,17,18,19,20,21,22,23,24,25,26,27,28^ Qualitative methods included validated patient-reported outcome measures (PROM; 10 studies),^23,24,25,26,29,30,31,32,33,34^ patient-reported severity of FT rated by the patient on a 3-point or 5-point scale (7 studies),^26,27,35,36,37,38,39^ qualitative interviews (4 studies),^28,40,41,42^ and other (2 studies).^43,44^ A study falling into multiple categories was analyzed in all categories.

**Table 1.** Summary of Study Characteristics^a^

Source	Study design	Outcome measures	Sample size	Quality assessment
Hastert et al,^12^ 2019, US	Cross-sectional	FT rate	436 Patients with BC	Good
Housser et al,^13^ 2013, Canada	Cross-sectional	FT rate	131 Patients with BC	Good
Jones et al,^14^ 2018, US	Cross-sectional	FT rate	127 Patients with BC	Fair
Kavosi et al,^15^ 2014, Iran^b^	Cross-sectional	FT rate	92 Households	Fair
The ACTION Study Group,^16^ 2015, Southeast Asia (Cambodia, Indonesia, Laos, Malaysia, Myanmar, the Philippines, Thailand, Vietnam)^b^	Prospective cohort	FT rate	2445 Patients with BC	Good
Knight et al,^17^ 2018, US	Cross-sectional	FT rate	612 Patients with BC	Good
Jain and Mukherjee,^18^ 2016, India^b^	Cross-sectional	FT rate	221 Patients with BC	Good
Nekhlyudov et al,^19^ 2016, US	Cross-sectional	FT rate	135 Patients with BC	Good
O’Neill et al,^4^ 2015, Haiti^b^	Cross-sectional	FT rate	61 Patients with BC	Fair
Palmer et al,^20^ 2018, US	Cross-sectional	FT rate	36 Patients with BC; 210 radiation oncologists	Fair
Rayce et al,^21^ 2008, Denmark	Retrospective cohort	FT rate	533 Patients with BC	Good
Subramanian et al,^22^ 2020, US	Cross-sectional	FT rate	830 Patients with BC	Good
Offodile et al,^23^ 2021, US	Cross-sectional	FT rate, PROM (COST)	571 Patients with BC	Good
Wan et al,^24^ 2021, US	Cross-sectional	FT rate, PROM (COST)	95 Patients with BC	Good
Irwin et al,^25^ 2014, US	Cross-sectional	FT rate, PROM (InCharge Financial Distress/Financial Well-Being score)	134 Patients with BC	Good
Goldberg et al,^26^ 2019, US	Cross-sectional	FT rate, patient-reported severity of FT, PROM (Cota Patient Assessed Symptom Score-7 item)	171 Patients with BC	Fair
Perry et al,^27^ 2019, US	Cross-sectional	FT rate, patient-reported severity of FT	309 Patients total (all assessed for financial strain), with a subset of 134 patients assessed for FT	Good
Wang et al,^28^ 2020, China^b^	Prospective cohort	FT rate, qualitative	44 Patients with BC	Fair
Asaad et al,^29^ 2020, US	Cross-sectional	PROM (COST)	195 Patients total (66 unilateral mastectomy, 129 contralateral prophylactic mastectomy)	Good
Jing et al,^30^ 2020, China^b^	Cross-sectional	PROM (COST)	166 Patients with BC	Good
Politi et al,^31^ 2021, US	Cross-sectional	PROM (COST)	395 Patients with BC	Good
Williams et al,^32^ 2020, US	Cross-sectional	PROM (COST)	84 Patients total, of whom 42 had low health literacy and 42 had high health literacy	Good
Dean et al,^33^ 2019, US	Prospective cohort	PROM (economic burden score)	40 Patients with BC	Fair
Meneses et al,^34^ 2012, US	Prospective cohort	PROM (Breast Cancer Finances Survey economic burden score)	132 Patients with BC	Good
Alexander et al,^35^ 2019, India^a^	Prospective cohort	Patient-reported severity of FT	378 Patients with BC	Good
Han et al,^36^ 2019, Korea	Cross-sectional	Patient-reported severity of FT	141 Patients with BC	Fair
Lauzier et al,^37^ 2008, Canada	Prospective cohort	Patient-reported severity of FT	459 Patients with BC	Good
Longo and Bereza,^38^ 2011, Canada	Cross-sectional	Patient-reported severity of FT	74 Patients with BC	Fair
Subramanian et al,^39^ 2019, Kenya^b^	Cohort	Patient-reported severity of FT	400 Patients with BC	Fair
Japhet et al,^40^ 2019, Nigeria^b^	Cross-sectional	Qualitative	22 Patients with BC	Poor
Rocque et al,^41^ 2019, US	Cross-sectional	Qualitative	20 Patients with BC; 11 oncologists	Poor
White-Means et al,^42^ 2020, US	Cross-sectional	Qualitative	5 Patients with BC	Poor
Gany et al,^43^ 2020, Egypt^b^	Cross-sectional	Quality of life measures	100 Patients with BC	Fair
Pezzin et al,^44^ 2009, US	Prospective cohort	Odds ratio	1890 Patients with BC	Good

Abbreviations: BC, breast cancer; COST, Comprehensive Score for Financial Toxicity; FT, financial toxicity; PROM, patient-reported outcome measure.

^a^ Characteristics and quality assessment of included studies are shown. All patients in the sample size listed are patients with BC; for studies where patients with BC are a subset of the entire study, it is specified that the sample listed refers to patients with BC only.

^b^ Denotes low- and middle-income countries.

### Statistical Analysis

Data analysis was performed from March to December 2022. Rate of FT, as defined and reported by each study, was used where available. In studies that measured patient-reported severity only, ratings were reported on a 3-point (1 = minimal, 2 = moderate, 3 = severe) or 5-point (1 = none, 2 = a little bit, 3 = somewhat, 4 = quite a bit, 5 = a lot) scale. To calculate a FT rate from these studies, we defined FT as a moderate to severe rating (score of 2-3 on a 3-point scale or 4-5 on a 5-point scale), because these cutoffs were used consistently across studies to determine FT.^26,27^ The percentage population exceeding the cutoff determined FT rate in these studies.

Statistical combination of the individual studies was performed to obtain a pooled estimate of the FT rate among patients with breast cancer in HICs and LMICs. We used a random-effects model by the DerSimonian and Laird method to account for heterogeneity between studies.^45^ Visual representation was achieved with a forest plot. The FT rate along with 95% CIs were calculated for quantitative estimation of FT and converted to percentages for better representation.

Heterogeneity was assessed using 2-sided χ^2^ test for homogeneity and *I*^2^ index for variability.^46^ Cutoff points for heterogeneity in the *I*^2^ index were as follows: *I*^2^ value less than 25%, low heterogeneity; 25% to 75%, moderate heterogeneity; and greater than 75%, high heterogeneity.^46^ If *P* < .10, the presence of heterogeneity was considered in the χ^2^ test. To account for heterogeneity, subgroup analysis was performed among HIC studies for different study quality ratings, because the number of studies within LMICs was limited (4 studies).

Study quality was assessed using a preexisting risk of bias tool.^47^ Fourteen criteria were independently assessed by 2 authors (C.A.W. and A.N.E.), and an overall quality rating was determined, corresponding to good, fair, or poor. Small study effects were assessed visually using funnel plot and statistically using Begg and Egger tests. The trim-and-fill method was used to account for any potential small study bias, given this was the source of asymmetry in the funnel plot. Statistical analysis was performed using Comprehensive Meta-Analysis statistical software version 4 (Biostat, Inc).

## Results

**Figure 1.**
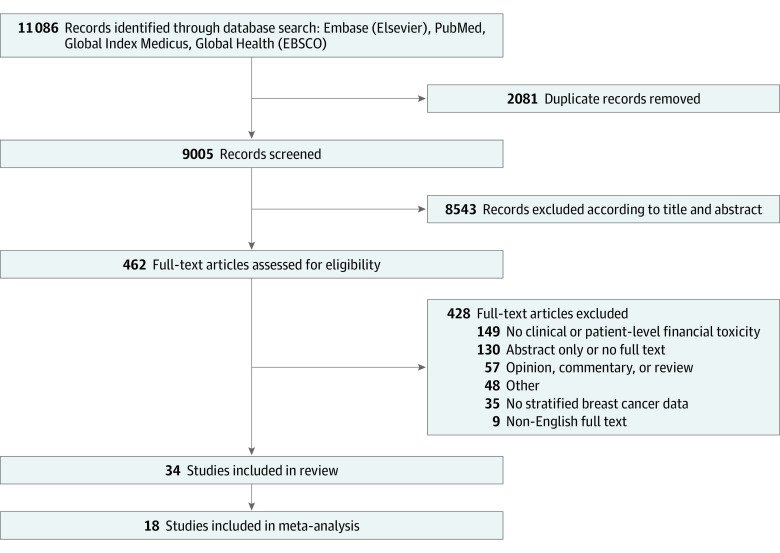
Flow Diagram of Included Studies

A total of 11 086 citations were retrieved. After deduplication, 9005 remained and were screened. Following title and abstract screening, 462 articles underwent full-text assessment, and 34 articles were included (Figure 1).

The sample size of included studies ranged from 5 to 2445 people (Table 1). All studies were published between 2008 and 2021. The majority were undertaken in HICs (24 studies), largely in the US (19 studies), followed by Canada (3 studies), South Korea (1 study), and Denmark (1 study). A total of 10 studies were from LMICs, including China (2 studies), India (2 studies), Haiti (1 study), Kenya (1 study), Nigeria (1 study), Egypt (1 study), Iran (1 study), and 1 study examining multiple Southeast Asian countries. Of the 34 studies, 26 were cross-sectional, 7 were prospective cohort, and 1 was retrospective cohort. Table 1 and Table 2 show additional details of the study characteristics.

**Table 2.** Summary of FT Measurement Modalities and Outcomes

Source	Measurement of FT^a^	Results
Hastert et al,^12^ 2019, US	Financial hardship was defined as having to refinance or take out second mortgage on home, sell home, sell stocks or other investments, or withdraw money from retirement accounts to pay for treatment; experiencing a decline in income since diagnosis; borrowing money; or being in debt due to cancer expenses. Limited care because of cost was defined as having to turn down treatment, skip medication doses, or decline seeing a physician because of cost.	48% of Patients with BC experienced financial hardship; 18.6% experienced limited care because of cost.
Housser et al,^13^ 2013, Canada	FT was defined as having trouble paying OOPC for BC. Patients were also asked whether OOPC created a lot of stress, created more stress than other things, or influenced treatment decisions.	10.1% of Patients with BC experienced FT. OOPC influenced treatment decisions in 9.6% of patients. OOPC created a lot of stress for 34.2% of patients, and 22.2% reported OOPC created more stress than other things.
Jones et al,^14^ 2018, US	Long-term cancer survivors completed the Medical Expenditure Panel Survey Center Survivorship Supplement to evaluate FT.	21.3% of Patients with BC reported experiencing financial difficulty.
Kavosi et al,^15^ 2014, Iran^b^	FT was defined as a ratio of household health expenditure to household capacity to pay >40%.	60.9% of Households with BC experienced FT.
The ACTION Study Group,^16^ 2015, Southeast Asia (Cambodia, Indonesia, Laos, Malaysia, Myanmar, the Philippines, Thailand, Vietnam)^b^	Financial catastrophe was defined as OOPC at 12 mo after diagnosis equal to or exceeding 30% of annual household income.	62% of Patients with BC survived but experienced financial catastrophe, 26% survived without experiencing financial catastrophe, and 12% of patients with BC died.
Knight et al,^17^ 2018, US	FT was defined as agreement with the statement, “You have to pay more for medical care than you can afford.”	26% of Patients with BC experienced FT.
Jain and Mukherjee,^18^ 2016, India^b^	Catastrophic health expenditure was defined as having OOPC (excluding reimbursement, if any) greater than or equal to 40% of total nonfood household expenditure.	84% of Households experienced catastrophic health expenditure.
Nekhlyudov et al,^19^ 2016, US	Financial burden was defined as having to borrow money, going into debt, making other financial sacrifices, or being unable to cover medical visit costs.	22% of Patients with BC experienced financial burden.
O’Neill et al,^4^ 2015, Haiti^b^	FT was defined as spending >40% of potential income on medical expenses.	68% of Patients who were estimate their annual income experienced FT; 89% of patients stopped working because of their illness, 49% used all of their savings to pay OOPC, and 52% of patients went into debt.
Palmer et al,^20^ 2018, US	FT was defined as loss of income, loss of job, loss of spouse, difficulty paying for meals, difficulty paying rent or mortgage, or difficulty paying for transportation.	27.8% of Patients with BC experienced FT.
Rayce et al,^21^ 2008, Denmark	FT was defined as moving 1 decile or more down the income distribution for the general population in the year following the year of diagnosis compared with the average income of the 2 y prior.	22.5% of Patients experienced FT.
Subramanian et al,^22^ 2020, US	FT was defined as forgoing medical treatment because of cost.	31.8% of Patients with BC forwent medical care because of cost, including missing a doctor’s appointment (14.7%), forgoing fertility preservation (11.9%), delaying or forgoing breast reconstruction (10.9%), missing follow-up imaging (9.46%), forgoing medication (7.3%) or taking less of a medication than prescribed (6.2%), or delaying or stopping treatment (4.2%).
Offodile et al,^23^ 2021, US	The COST was used to measure FT. The score ranges from 0 to 44, with a lower score representing higher FT. FT was defined as having a COST ≤30.	51.3% of patients experienced FT.
Wan et al,^24^ 2021, US	The COST was used to measure FT. The score ranges from 0 to 44, with a lower score representing higher FT. Severe FT was defined as a COST ≤10.	Median (IQR) COST was 22 (16-29).; 10.5% of patients experienced severe FT.
Irwin et al,^25^ 2014, US	The InCharge Financial Distress/Financial Well-Being score was used to assess FT. A score <6 was defined as moderate or greater financial distress, and a score of ≥6 was defined as low or no financial distress.	44% of Patients experienced moderate or greater financial distress.
Goldberg et al,^26^ 2019, US	A survey question from the Cota Patient Assessed Symptom Score-7 item asked patients to rate the impact of BC on their financial well-being (none, minimal, moderate, significant, severe). A rating of moderate, significant, or severe was defined as experiencing FT.	39% of Patients with BC experienced FT.
Perry et al,^27^ 2019, US	Financial strain was defined as being unable to afford 1 or more of the following 4 categories: (1) food and housing, (2) clothing, medicine, and home repairs, (3) going out for a meal and entertainment, or (4) a week-long vacation. Patients were asked to rate their agreement with the statement, “I have to pay more for my medical care than I can afford” on a 5-point scale (strongly disagree, disagree, neutral, agree, strongly agree) and FT was defined at a rating of agree or strongly agree.	37.5% of Women reported financial strain; 26.1% reported FT.
Wang et al,^28^ 2020, China^b^	The use of expressive writing from Chinese BC survivors was used to assess their experiences. For 30 min a week over a 3-wk period, participants wrote about decision-making, family influences, and cultural influences. The writings were qualitatively analyzed.	34.1% of Patients mentioned financial burden as a theme.
Asaad et al,^29^ 2020, US	The COST was used to measure FT. The score ranges from 0 to 44, with a lower score representing higher FT.	Mean (SD) COST was 26.71 (10.87) among unilateral mastectomy patients and 28.64 (11.00) among contralateral prophylactic mastectomy patients (*P* = .53).
Jing et al,^30^ 2020, China^b^	The COST was used to measure FT. The score ranges from 0 to 44, with a lower score representing higher FT.	Mean (SD) COST was 21.2 (8.1), and median COST was 22.5.
Politi et al,^31^ 2021, US	A 4-question validated subset of the COST was used to assess FT at 1 wk, 12 wk, and 1 y after diagnosis. Scores ranged from 0 to 16.	Mean (SD) scores were 6.4 (4.2) at 1 wk, 6.2 (4.3) at 12 wk, and 6.2 (4.8) at 1 y.
Williams et al,^32^ 2020, US	The COST was used to measure FT. The score ranges from 0 to 44, with a lower score representing higher FT.	Median (IQR) COST was 25 (17-31) among high health literacy patients. Median (IQR) COST was 22 (16-25) among low healthy literacy patients.
Dean et al,^33^ 2019, US	An economic burden score (ranging from 0 to 12) was used to measure the financial impact of BC.	Mean (SD) economic burden score was 2.5 (4).
Meneses et al,^34^ 2012, US	Economic hardship was assessed using the 19 economic burden items on the Breast Cancer Finances Survey Inventory at baseline, 3 mo, and 6 mo.	Mean of 2.94 economic burden items (median, 2; range, 0-11) at baseline, 2.45 items (mean, 1; range, 0-13) at 3 mo, and 2.25 items (mean, 1; range, 0-14) at 6 mo.
Alexander et al,^35^ 2019, India^b^	Patients without health insurance were asked to rate financial difficulty on a scale (mild, moderate, severe).	29% of Patients had health insurance. Of the remainder, 28% of patients experienced mild, 30% experienced moderate, and 13% experienced severe financial difficulty.
Han et al,^36^ 2019, Korea	Patients were asked to rate their financial burden on a scale (none, moderate, substantial-to-heavy).	16% of Patients reported no burden, 51% of patients experienced moderate burden, and 33% of patients experienced substantial-to-heavy burden.
Lauzier et al,^37^ 2008, Canada	Patients were asked to rate the costliness of BC on a 5-point scale (not at all costly to very costly)	14.7% of Patients reported that BC had been very costly.
Longo and Bereza,^38^ 2011, Canada	Patients were asked to rate the degree to which OOPC represented a financial burden on a 5-point scale (not at all, slightly, somewhat, significant but manageable, unmanageable).	31% of Patients with BC reported that OOPC were a significant or unmanageable burden, compared with 17% of patients without BC (*P* = .01).
Subramanian et al,^39^ 2019, Kenya^b^	Patients were asked to rate the financial impact of BC on a 5-point scale (not much at all, a little, somewhat, quite a bit, very much).	85% of Patients rated financial impact as “very much,” 6.5% rated the impact as “quite a bit,” 3% rated the impact as “somewhat,” 2.8% rated “a little,” and 1.8% rated “not much at all.”
Japhet et al,^40^ 2019, Nigeria^b^	Patients were interviewed about the impact of BC on many aspects of their lives, including financial burden.	The financial burden of both direct and indirect costs was highlighted as a theme throughout several patient interviews.
Rocque et al,^41^ 2019, US	Patients were interviewed about the impact of BC on their lives.	Several surveyed patients mentioned cost or FT as a theme in their interviews.
White-Means et al,^42^ 2020, US	Patients with BC were interviewed about the impact of BC on their lives.	Financial burden was mentioned as a theme throughout many interviews.
Gany et al,^43^ 2020, Egypt^b^	Patients were asked to complete the US Department of Agriculture Food Security Assessment as well as other survey questions about financial assistance needs, using savings to pay for cancer treatment, or not having savings.	66% of Patients needed financial assistance; 80% of patients with greater financial needs had difficulty affording medications; 47.5% of patients were food-insecure, and almost 33% reported difficulty in affording transportation costs.
Pezzin et al,^44^ 2009, US	Financial difficulty was defined as a rating of “somewhat difficult” or “very difficult” to pay medical bills including prescribed medications when rated on a 5-point Likert scale. The likelihood of experiencing financial difficulty was compared between women taking aromatase inhibitors and those taking tamoxifen.	Women taking only aromatase inhibitors were more likely to experience financial difficulty than those taking only tamoxifen (OR, 1.4; 95% CI, 1.1-1.7). Having no drug coverage (OR, 4.5; 95% CI, 3.3-5.9) or partial drug coverage (OR, 3.6; 95% CI, 2.8-4.5) was associated with increased financial difficulty compared with full coverage.

Abbreviations: BC, breast cancer; COST, Comprehensive Score for Financial Toxicity; FT, financial toxicity; OOPC, out-of-pocket costs; OR, odds ratio.

^a^ FT measurement and results of included studies. All results are calculated on the basis of total number of patients with BC in the study unless otherwise specified.

^b^ Denotes low- and middle-income countries.

Most studies (20 studies [58.8%]) reported health insurance status. Universal public insurance was available in China, South Korea, Kenya, and Canada. A mix of private and public insurance was seen in the US and Iran. In India and Southeast Asia, few patients had insurance, and most paid entirely out-of-pocket. Income was reported in most studies (24 studies [70.6%]). Reporting was heterogeneous, with highly variable definitions of the lowest reported income bracket. Definitions ranged from descriptive including “being below the poverty line for the country” or “low income,” to quantifiable including earning less than 25% of mean national household income or earning less than a specific number (ranging from <$12 000 US to <$50 000 US annual household income). Overall, 24.3% fell into the lowest income bracket. Among US studies reporting race,^12,14,17,19,20,22,23,24,25,27,29,31,32,33,34,41,44^ an average of 73.7% of patients were White. Race was not reported in other countries. All studies evaluated FT during diagnosis and treatment for breast cancer, and none did so during screening.

Many studies reported a FT rate (18 studies); there was significant heterogeneity in the definition of FT across these studies. Five studies had specific numerical criteria for defining FT. This included medical cost exceeding 40% of household capacity to pay or potential income (3 studies),^4,15,18^ OOPC exceeding 30% of annual household income (1 study),^16^ and moving more than 1 decile down the national income distribution (1 study).^21^ Four studies used PROM instruments, further described later.^23,24,25,26^ Five studies used patient self-identification based on subjective financial difficulty criteria. Here, FT was defined as an affirmative answer to having financial difficulty,^39^ trouble paying medical bills,^13^ or having to pay more for medical care than is affordable,^17,27^ or if patients brought up financial burden as a theme in qualitative interviews.^28^ In 4 studies, FT was defined according to patients’ report of specific, objective financial consequences of care, including losing income or a job; change in marital status; having to borrow money or go into debt; having difficulty paying for food, rent, or transportation^12,19,20^; or having to forgo any type of medical treatment because of cost.^22^

Ten studies used validated PROM instruments. Of these, 4 calculated a FT rate. These 4 studies used the Comprehensive Score for Financial Toxicity (COST),^48^ though with varying cutoff scores to define FT (2 studies),^23,24^ the InCharge Financial Distress/Financial Well-Being Scale (1 study),^25,45^ and the Cota Patient Assessed Symptom Score-7 instrument (1 study).^26^ The COST is a validated instrument with a score range of 0 to 44, with lower scores associated with greater FT.^48^ Economic burden scores were calculated using the Breast Cancer Finances Survey or another validated tool containing questions about crucial aspects of cancer-related economic burden.^33,34^

Six studies^27,35,36,37,38,39^ used patient-reported severity of financial burden rated on a 3-point or 5-point scale. These studies asked patients to rate the degree of financial impact (1 study), difficulty (2 studies), or burden (3 studies) posed by breast cancer care. Four qualitative studies^28,40,41,42^ offered representative quotations describing patient experiences, including high OOPCs, financial worry, and negative impact on patient and family financial well-being. Two other studies^43,44^ used varied methods. Pezzin et al^44^ defined FT as a rating of somewhat difficult or very difficult on a question about difficulty paying medical bills when comparing 2 medication groups and compared the odds of FT among individuals in 2 treatment groups. Gany et al^43^ reported the impact of financial consequences on several quality of life measures, such as food insecurity, financial assistance, and savings.

**Figure 2.**
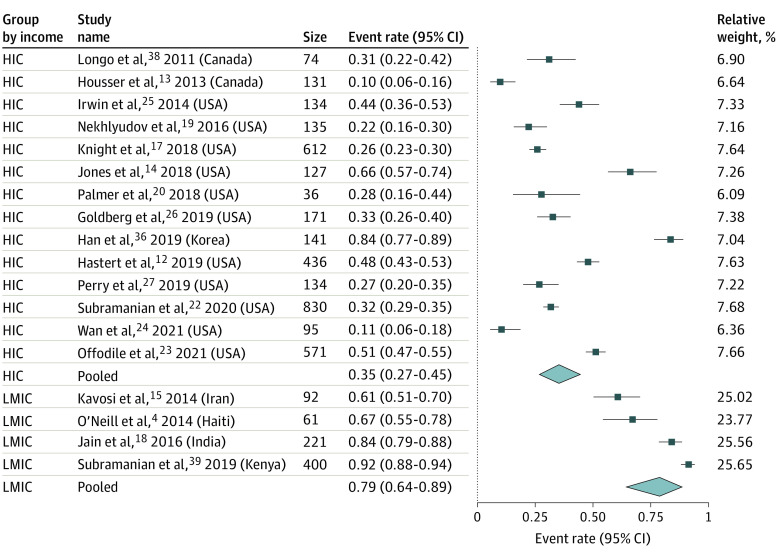
Forest Plot for Financial Toxicity Rate, Subgrouped by High-Income Countries (HICs) and Low-to-Middle Income Countries (LMICs)

Eighteen studies^4,12,13,14,15,17,18,19,20,22,23,24,25,26,27,36,38,39^ were eligible for meta-analysis, 14 from HICs and 4 from LMICs. The rate of FT among patients with breast cancer was 35.3% (95% CI, 27.3%-44.4%; *I*^2^ = 95.95%; *P* for heterogeneity, <.001) in HICs and 78.8% (95% CI, 60.4%-90.0%; *I*^2^ = 94.78%; *P* for heterogeneity, <.001) in LMICs (*P* < .001 for HICs vs LMICs) (Figure 2). A subgroup analysis based on study quality ratings was performed among 14 studies focusing on FT in HICs. The rate of FT among patients with breast cancer was 49.9% (95% CI, 34.7%-65.1%; *I*^2^ = 96.0%; *P* for heterogeneity, <.001) in studies rated fair in quality assessment and 28.5% (95% CI, 20.2%-38.6%; *I*^2^ = 95.7%; *P* for heterogeneity, <.001) in studies rated good in quality assessment (*P* < .001 for fair vs good quality assessment) (eFigure 1 in Supplement 1).The funnel plot visually showed slight asymmetry toward the right of the pooled point estimate for HIC studies; however, both Begg rank correlation test and Egger linear regression test indicated no small study bias. Conditioning on small study being the source of asymmetry, the trim-and-fill method revealed an overall imputed rate estimate of 42.7% (95% CI, 33.7%-52.3%), which was not materially different from 35.0% (95% CI, 27.0%-45.9%) (eFigure 2 in Supplement 1).

FT in studies without a reported or calculatable FT rate varied. The full COST was reported in 4 studies. Mean COST ranged from 21.2 in Jin et al^30^ to 28.64 in Asaad et al,^29^ and the median COST ranged from 22 in Wan et al^24^ to 25 in Williams et al.^32^ One study^31^ used a 4-question subset of the COST scored out of 16, with a mean (SD) score of 6.2 (4.8) 1 year after breast surgery. An alternate PROM used in 2 studies^33,34^ was an economic burden score, with a mean of 2.25 to 2.94 economic burden items per patient at various times after diagnosis. The qualitative studies used interviews with patients (3 studies)^28,40,41^ and/or breast cancer assistance programs representatives (1 study)^42^ to understand FT impact. One study using odds ratios to compare FT between 2 adjuvant treatment groups found that patients receiving aromatase inhibitors, compared with those receiving tamoxifen, experienced higher FT rates (odds ratio, 1.4), although absolute FT rates were not reported.^44^ The study assessing quality of life measures found that 47.5% of patients were food insecure, 66.0% needed financial assistance, 34.0% used savings to pay for treatment, and 41.2% lacked savings altogether.^43^

Race and FT had no clear association.^12,14,17,19,20,22,23,24,25,27,28,29,31,32,33,34,41,44^ Patients’ education level^4,12,13,14,15,16,17,18,19,20,21,22,23,24,25,27,28,29,30,32,33,34,35,37,39,41,42,43^ or insurance status^12,13,15,16,18,19,20,22,23,24,25,29,30,31,32,33,35,36,39,44^ were related to the income status of the country. The employment status of the patient was related to age, with older patient populations having more retirees, as well as to prevalence of homemakers compared with women working outside the home.^12,13,17,18,21,22,23,25,28,29,30,34,35,36,37,39,42,43^ No clear association was found between these factors and FT. Few studies reported comorbidities (8 studies)^12,19,22,23,30,33,37,44^ or urban vs rural status (6 studies)^13,24,30,34,35,39^; thus, conclusions could not be drawn. Cancer stage and treatments were extremely heterogeneous across studies; no clear association was found between either factor and FT.

## Discussion

This systematic review and meta-analysis found a high rate of FT among individuals undergoing breast cancer treatment globally. Overall, 35.3% of patients in HIC studies and 78.8% of patients in LMIC studies experienced FT. To our knowledge, this study is the first to systematically characterize the global burden of FT. Typical FT rates across all health conditions in LMICs range from 6% to 12%.^49,50,51^ Our study demonstrates that FT prevalence among patients with breast cancer is substantially higher, with vulnerability compounded in LMICs.

A high global burden of FT due to malignant neoplasms is unsurprising given the need for expensive, multidisciplinary treatment at specialized facilities, prolonged follow-up, transportation costs, and time off work.^37^ This burden has often been associated with adverse clinical outcomes, including mortality.^52,53^ However, the financial burden of breast cancer is even greater than those for other cancers, with substantially higher OOPC than colorectal, lung, and prostate combined.^38^ At a systems level, the Lancet Oncology Commission demonstrated that breast cancer was the most expensive cancer in the US in 2010, accounting for US $16.5 billion, or 13% of all cancer-related spending.^53,54^ Individually, direct medical costs of breast cancer care range up to US $100 000.^55^

Gaps remain in determining the cause of increased costs and FT for breast cancer specifically. In HICs, this may be attributable to many novel and costly cancer care interventions, services overutilization, increased willingness to pay, and varying insurance coverage.^53^ Individuals in LMICs encounter delayed diagnoses due to limited access to screening and high-quality diagnostic services, leading to higher prevalence of later-stage diagnoses requiring more extensive treatments. Lower baseline income, limited insurance coverage, and greater travel distances for specialized cancer care also contribute.^56,57^ The high incidence of FT among patients with breast cancer, especially in LMICs and compared with other diseases, presents an important target for cancer control policies and programs. Because higher FT is associated with worse patient outcomes, addressing FT is an important intervention to reduce the substantial, growing rates of breast cancer mortality in LMICs. Highest priority patient populations have low baseline education and socioeconomic status, lack health insurance, and live in low-resource areas. These populations are not exclusive to LMICs, but also exist in low-resource HIC areas. On the basis of our findings, we recommend 4 potential intervention areas to reduce FT and improve outcomes among patients with breast cancer: targeted educational campaigns to raise awareness about the signs and symptoms of breast cancer and the importance of early diagnosis and treatment; expansion of health care coverage to minimize direct medical OOPC^58^; programs to assist with direct nonmedical and indirect costs, such as transportation to and lodging near treatment centers, childcare, and other family expenses incurred as a result of treatment; and funneling resources toward improving screening, referral, and treatment infrastructure for breast cancer care. Our data further suggest that there are lower FT rates in countries with universal health coverage, demonstrating efficacy of this health care policy approach. However, there are no comparative studies, and conclusive outcomes cannot be determined with existing data.

Our study highlights the need for more studies assessing FT. There are limited LMIC data, and most HIC data are from the US; fully understanding the burden in different regions and contexts is integral to executing potential solutions. There is also a clear need to standardize FT measurement to improve generalizability across studies. Definitions for FT ranged from patient reports to a single survey question to numerical expenditure-based thresholds. Other studies used patient-reported experiences of FT without providing a quantifiable estimate. We recommend a combined approach using both qualitative and quantitative variables to measure FT. Quantitatively, FT can be measured as catastrophic health expenditure, defined using the total OOPC as a percentage of household income or discretionary (nonfood) expenditure. The most widely used metric of FT is medical cost greater than 40% of total household nonfood expenditure.^59^ Qualitative measures include validated PROMs on food scarcity, joblessness, and childhood education.

### Limitations

Our study has important limitations. We were limited to English-language articles because of personnel available to conduct the study; however, we still captured a wide range of international studies. There was significant heterogeneity between studies in FT definition and demographic data reporting; in addition, income, rural vs urban status, and FT definitions were not reported uniformly. Nevertheless, we explored country income and study quality as sources of heterogeneity when possible. Other inconsistently reported variables were employment status, stage of breast cancer, comorbidities, and treatments. Consistent reporting of these variables is necessary to examine their potential contribution to FT and facilitate geographic comparisons. FT patterns and interventions vary with health care systems, and in LMICs particularly, financial planning is integral to treatment planning and national goals. Our study did not account for different health care systems or control for health care–dedicated gross domestic product. Our calculation, therefore, is an important global overview of FT among patients with breast cancer and represents a starting point for future analyses.

## Conclusions

FT is a substantial burden of cancer treatment among patients with breast cancer. The incidence of breast cancer–related FT is 78.8% in LMICs and 35.3% in HICs. The definition and measurement of FT varied across studies, and reporting of covariates was inconsistent. Further efforts, ideally led by transnational organizations, are required to thoroughly characterize FT burden in a standardized fashion across different settings. Policy interventions to improve breast cancer education, expand health care coverage, enhance patient support for nonmedical costs, and invest in health care infrastructure could improve both financial and health outcomes among patients with breast cancer at risk for FT.

## Notes

Supplement 1. **eAppendix. **Search Strategy **eFigure 1.** Forest Plot for Financial Toxicity Rate, Subgrouped by Study Quality Ratings Among High-Income Countries (HICs) **eFigure 2.** Funnel Plot of Standard Error by Logit Event Rate for Studies Reporting High-Income Countries (HICs)

Supplement 2.
Data Sharing Statement
