# Supplementary material for: Financial Toxicity Among Patients With Breast Cancer Worldwide: A Systematic Review and Meta-analysis
Source: JAMA Netw Open. 2023 Feb 8;6(2):e2255388. doi: 10.1001/jamanetworkopen.2022.55388 (PMC9909501; doi:10.1001/jamanetworkopen.2022.55388)
Supplement: Supplement 1. — eAppendix. Search Strategy eFigure 1. Forest Plot for Financial Toxicity Rate, Subgrouped by Study Quality Ratings Among High-Income Countries (HICs) eFigure 2. Funnel Plot of Standard Error by Logit Event Rate for Studies Reporting High-Income Countries (HICs) [file jamanetwopen-e2255388-s001.pdf]

Supplemental Online Content

Ehsan AN, Wu CA, Minasian A, et al. Financial toxicity among patients with breast cancer worldwide: a systematic review and meta-analysis. *JAMA Netw Open*. 2023;6(2):e2255388. doi:10.1001/jamanetworkopen.2022.55388

eAppendix. Search Strategy
eFigure 1. Forest Plot for Financial Toxicity Rate, Subgrouped by Study Quality Ratings Among High-Income Countries (HICs)
eFigure 2. Funnel Plot of Standard Error by Logit Event Rate for Studies Reporting High-Income Countries (HICs)

This supplemental material has been provided by the authors to give readers additional information about their work.

© 2023 Ehsan AN et al. *JAMA Network Open.*

eAppendix. Search Strategy

Wu Out of Pocket Finances Breast Cancer Search Strategy
Last Updated: February 9, 2021

What is the out-of-pocket financial burden of breast cancer care in high-, middle-, and low-income countries, and what interventions have been implemented to address this burden?

Citation Pull: February 9, 2021

PubMed: 2240
Embase (Elsevier): 8231
Global Index Medicus: 61
Global Health (EBSCO): 554

Direct import into Covidence
Embase – 8176 (55 duplicates)
PubMed – 547 (1693 duplicates)
Global Index Medicus – 46 (15 duplicates)
Global Health – 239 (315 duplicates)

Total number of articles to review in Title/Abstract Screening: 9008

Concept 1: Breast Cancer
Synonyms
breast cancer* OR breast neoplasm* OR breast tumour* OR breast tumor* OR breast carcinoma* OR carcinoma of the breast OR carcinoma in situ OR mammary cancer* OR cancer of breast OR cancer of the breast

Concept 2: Out of Pocket Financial Burden
Synonyms
Financial Stress* OR Financial Pressure* OR Financial Hardship* OR Financial Challenges* OR Financial burden* OR health expenditure* OR direct expenditure* OR out-of-pocket expenditure* OR out-of pocket expenditure* OR out of pocket expenditure* OR out-of-pocket expense* OR out-of pocket expense* OR out of pocket expense* OR out-of-pocket cost* OR out-of pocket cost* OR out of pocket cost* OR out-of-pocket payment* OR out-of pocket payment* OR out of pocket payment* OR out-of-pocket spending OR out-of pocket spending OR out of pocket spending OR indirect expenditure* OR expenditure* OR indirect cost* OR fear of cost* OR cost of treatment OR capital expenditure* OR direct service cost* OR health expenditure* OR healthcare expenditure* OR health care expenditure* OR health care cost* OR healthcare cost* OR medical care cost* OR health cost* OR treatment cost*

PubMed

© 2023 Ehsan AN et al. *JAMA Network Open.*

Concept 1: Breast Cancer
Controlled Vocabulary
"Breast Neoplasms"[Mesh]

Concept 2: Out-of-Pocket Expenses
Controlled Vocabulary
"Health Care Costs"[Mesh] OR "Financial Stress"[Mesh] OR "Health Expenditures"[Mesh]

Concept 1 AND Concept 2

(("Breast Neoplasms"[Mesh]) OR (breast cancer*[Title/Abstract] OR breast neoplasm*[Title/Abstract] OR breast tumour*[Title/Abstract] OR breast tumor*[Title/Abstract] OR breast carcinoma*[Title/Abstract] OR carcinoma of the breast[Title/Abstract] OR carcinoma in situ[Title/Abstract] OR mammary cancer*[Title/Abstract] OR cancer of breast[Title/Abstract] OR cancer of the breast[Title/Abstract])) AND (("Health Care Costs"[Mesh] OR "Financial Stress"[Mesh] OR "Health Expenditures"[Mesh]) OR (Financial Stress*[Title/Abstract] OR Financial Pressure*[Title/Abstract] OR Financial Hardship*[Title/Abstract] OR Financial Challenges*[Title/Abstract] OR Financial burden*[Title/Abstract] OR health expenditure*[Title/Abstract] OR direct expenditure*[Title/Abstract] OR out-of-pocket expenditure*[Title/Abstract] OR out-of pocket expenditure*[Title/Abstract] OR out of pocket expenditure*[Title/Abstract] OR out-of-pocket expense*[Title/Abstract] OR out-of pocket expense*[Title/Abstract] OR out of pocket expense*[Title/Abstract] OR out-of-pocket cost*[Title/Abstract] OR out-of pocket cost*[Title/Abstract] OR out of pocket cost*[Title/Abstract] OR out-of-pocket payment*[Title/Abstract] OR out-of pocket payment*[Title/Abstract] OR out of pocket payment*[Title/Abstract] OR out-of-pocket spending[Title/Abstract] OR out-of pocket spending[Title/Abstract] OR out of pocket spending[Title/Abstract] OR indirect expenditure*[Title/Abstract] OR expenditure*[Title/Abstract] OR indirect cost*[Title/Abstract] OR fear of cost*[Title/Abstract] OR cost of treatment[Title/Abstract] OR capital expenditure*[Title/Abstract] OR direct service cost*[Title/Abstract] OR health expenditure*[Title/Abstract] OR healthcare expenditure*[Title/Abstract] OR health care expenditure*[Title/Abstract] OR health care cost*[Title/Abstract] OR healthcare cost*[Title/Abstract] OR medical care cost*[Title/Abstract] OR health cost*[Title/Abstract] OR treatment cost*[Title/Abstract]))

Results: 2240

EMBASE (Elsevier)
Concept 1: Breast Cancer
Controlled Vocabulary
'breast cancer'/exp

Concept 2: Out-of-pocket expenses
Controlled Vocabulary

© 2023 Ehsan AN et al. *JAMA Network Open.*

'financial stress'/exp OR 'financial burden'/exp OR 'out of pocket cost'/exp OR 'out of pocket expenditure'/exp OR 'out of pocket payment'/exp OR 'out of pocket spending'/exp OR 'treatment cost'/exp OR 'financial hardship'/exp OR 'health care cost'/exp

Concept 1 AND Concept 2
('breast cancer'/exp OR ((breast* NEAR/3 (cancer* OR tumor* OR tumour* OR neoplasm* OR adenocarcinoma* OR carcinoma* OR malignan* OR 'in situ')):ti,ab,kw) OR 'breast cancer*':ti,ab,kw OR 'breast neoplasm*':ti,ab,kw OR 'breast tumour*':ti,ab,kw OR 'breast tumor*':ti,ab,kw OR 'breast carcinoma*':ti,ab,kw OR 'carcinoma of the breast':ti,ab,kw OR 'carcinoma in situ':ti,ab,kw OR 'mammary cancer*':ti,ab,kw OR 'cancer of breast':ti,ab,kw OR 'cancer of the breast':ti,ab,kw) AND ('financial stress'/exp OR 'financial burden'/exp OR 'out of pocket cost'/exp OR 'out of pocket expenditure'/exp OR 'out of pocket payment'/exp OR 'out of pocket spending'/exp OR 'treatment cost'/exp OR 'financial hardship'/exp OR 'health care cost'/exp OR 'financial stress*':ti,ab,kw OR 'financial pressure*':ti,ab,kw OR 'financial hardship*':ti,ab,kw OR 'financial challenges*':ti,ab,kw OR 'financial burden*':ti,ab,kw OR 'direct expenditure*':ti,ab,kw OR 'out-of-pocket expenditure*':ti,ab,kw OR 'out-of pocket expenditure*':ti,ab,kw OR 'out of pocket expenditure*':ti,ab,kw OR 'out-of-pocket expense*':ti,ab,kw OR 'out-of pocket expense*':ti,ab,kw OR 'out of pocket expense*':ti,ab,kw OR 'out-of-pocket cost*':ti,ab,kw OR 'out-of pocket cost*':ti,ab,kw OR 'out of pocket cost*':ti,ab,kw OR 'out-of-pocket payment*':ti,ab,kw OR 'out-of pocket payment*':ti,ab,kw OR 'out of pocket payment*':ti,ab,kw OR 'out-of-pocket spending':ti,ab,kw OR 'out-of pocket spending':ti,ab,kw OR 'out of pocket spending':ti,ab,kw OR 'indirect expenditure*':ti,ab,kw OR expenditure*:ti,ab,kw OR 'indirect cost*':ti,ab,kw OR 'fear of cost*':ti,ab,kw OR 'cost of treatment':ti,ab,kw OR 'capital expenditure*':ti,ab,kw OR 'direct service cost*':ti,ab,kw OR 'health expenditure*':ti,ab,kw OR 'healthcare expenditure*':ti,ab,kw OR 'health care expenditure*':ti,ab,kw OR 'health care cost*':ti,ab,kw OR 'healthcare cost*':ti,ab,kw OR 'medical care cost*':ti,ab,kw OR 'health cost*':ti,ab,kw OR 'treatment cost*':ti,ab,kw)

Results 8231

Global Health (EBSCO)

Concept 1: Breast Cancer
Controlled Vocabulary
DE "breast cancer"

Concept 2: Out-of-pocket expenses
Controlled Vocabulary
DE "health care costs"

Concept 1 AND Concept 2

(DE "breast cancer" OR AB (breast cancer* OR breast neoplasm* OR breast tumour* OR breast tumor* OR breast carcinoma* OR carcinoma of the breast OR carcinoma in situ OR mammary cancer* OR cancer of breast OR cancer of the breast) OR TI (breast cancer* OR breast neoplasm* OR breast tumour* OR breast tumor* OR breast carcinoma* OR carcinoma of the

© 2023 Ehsan AN et al. *JAMA Network Open.*

breast OR carcinoma in situ OR mammary cancer* OR cancer of breast OR cancer of the breast)) AND (DE "health care costs" OR AB (Financial Stress* OR Financial Pressure* OR Financial Hardship* OR Financial Challenges* OR Financial burden* OR health expenditure* OR direct expenditure* OR out-of-pocket expenditure* OR out-of pocket expenditure* OR out of pocket expenditure* OR out-of-pocket expense* OR out-of pocket expense* OR out of pocket expense* OR out-of-pocket cost* OR out-of pocket cost* OR out of pocket cost* OR out-of-pocket payment* OR out-of pocket payment* OR out of pocket payment* OR out-of-pocket spending OR out-of pocket spending OR out of pocket spending OR indirect expenditure* OR expenditure* OR indirect cost* OR fear of cost* OR cost of treatment OR capital expenditure* OR direct service cost* OR health expenditure* OR healthcare expenditure* OR health care expenditure* OR health care cost* OR healthcare cost* OR medical care cost* OR health cost* OR treatment cost*) OR TI (Financial Stress* OR Financial Pressure* OR Financial Hardship* OR Financial Challenges* OR Financial burden* OR health expenditure* OR direct expenditure* OR out-of-pocket expenditure* OR out-of pocket expenditure* OR out of pocket expenditure* OR out-of-pocket expense* OR out-of pocket expense* OR out of pocket expense* OR out-of-pocket cost* OR out-of pocket cost* OR out of pocket cost* OR out-of-pocket payment* OR out-of pocket payment* OR out of pocket payment* OR out-of-pocket spending OR out-of pocket spending OR out of pocket spending OR indirect expenditure* OR expenditure* OR indirect cost* OR fear of cost* OR cost of treatment OR capital expenditure* OR direct service cost* OR health expenditure* OR healthcare expenditure* OR health care expenditure* OR health care cost* OR healthcare cost* OR medical care cost* OR health cost* OR treatment cost*))
Results 554

Global Index Medicus

Concept 1 AND Concept 2

(tw:("breast cancer*" OR "breast neoplasm*" OR "breast tumour*" OR "breast tumor*" OR "breast carcinoma*" OR "carcinoma of the breast" OR "carcinoma in situ" OR "mammary cancer*" OR "cancer of breast " OR "cancer of the breast")) AND (tw:("Financial Stress*" OR "Financial Pressure*" OR "Financial Hardship*" OR "Financial Challenges*" OR "Financial burden*" OR "health expenditure*" OR "direct expenditure*" OR "out-of-pocket expenditure*" OR "out-of pocket expenditure*" OR "out of pocket expenditure*" OR "out-of-pocket expense*" OR "out-of pocket expense*" OR "out of pocket expense*" OR "out-of-pocket cost*" OR "out-of pocket cost*" OR "out of pocket cost*" OR "out-of-pocket payment*" OR "out-of pocket payment*" OR "out of pocket payment*" OR "out-of-pocket spending" OR "out-of pocket spending" OR "out of pocket spending" OR "indirect expenditure*" OR "expenditure*" OR "indirect cost*" OR "fear of cost*" OR "cost of treatment" OR "capital expenditure*" OR "direct service cost*" OR "health expenditure*" OR "healthcare expenditure*" OR "health care expenditure*" OR "health care cost*" OR "healthcare cost*" OR "medical care cost*" OR "health cost*" OR "treatment cost*"))
Results 61

© 2023 Ehsan AN et al. *JAMA Network Open.*

PubMed
Concept 1: Breast Cancer
Controlled Vocabulary
"Breast Neoplasms"[Mesh]

Synonyms
breast cancer* OR breast neoplasm* OR breast tumour* OR breast tumor* OR breast carcinoma* OR carcinoma of the breast OR carcinoma in situ OR mammary cancer* OR cancer of breast OR cancer of the breast

Syntax
("Breast Neoplasms"[Mesh]) OR (breast cancer*[Title/Abstract] OR breast neoplasm*[Title/Abstract] OR breast tumour*[Title/Abstract] OR breast tumor*[Title/Abstract] OR breast carcinoma*[Title/Abstract] OR carcinoma of the breast[Title/Abstract] OR carcinoma in situ[Title/Abstract] OR mammary cancer*[Title/Abstract] OR cancer of breast[Title/Abstract] OR cancer of the breast[Title/Abstract])

Concept 2: Out-of-Pocket Financial Burden

Controlled Vocabulary
"Financial Stress"[Mesh] OR "Health Expenditures"[Mesh]

Synonyms
Financial Stress* OR Financial Pressure* OR Financial Hardship* OR Financial Challenges* OR Financial burden* OR health expenditure* OR direct expenditure* OR out-of-pocket expenditure* OR out-of pocket expenditure* OR out of pocket expenditure* OR out-of-pocket expense* OR out-of pocket expense* OR out of pocket expense* OR out-of-pocket cost* OR out-of pocket cost* OR out of pocket cost* OR out-of-pocket payment* OR out-of pocket payment* OR out of pocket payment* OR out-of-pocket spending OR out-of pocket spending OR out of pocket spending OR indirect expenditure* OR expenditure* OR indirect cost* OR fear of cost* OR cost of treatment

Syntax
("Financial Stress"[Mesh] OR "Health Expenditures"[Mesh]) OR (Financial Stress*[Title/Abstract] OR Financial Pressure*[Title/Abstract] OR Financial Hardship*[Title/Abstract] OR Financial Challenges*[Title/Abstract] OR Financial burden*[Title/Abstract] OR health expenditure*[Title/Abstract] OR direct expenditure*[Title/Abstract] OR out-of-pocket expenditure*[Title/Abstract] OR out-of pocket expenditure*[Title/Abstract] OR out of pocket expenditure*[Title/Abstract] OR out-of-pocket expense*[Title/Abstract] OR out-of pocket expense*[Title/Abstract] OR out of pocket expense*[Title/Abstract] OR out-of-pocket cost*[Title/Abstract] OR out-of pocket cost*[Title/Abstract] OR out of pocket cost*[Title/Abstract] OR out-of-pocket payment*[Title/Abstract] OR out-of pocket payment*[Title/Abstract] OR out of pocket

© 2023 Ehsan AN et al. *JAMA Network Open.*

payment*[Title/Abstract] OR out-of-pocket spending[Title/Abstract] OR out-of pocket spending[Title/Abstract] OR out of pocket spending[Title/Abstract] OR indirect expenditure*[Title/Abstract] OR expenditure*[Title/Abstract] OR indirect cost*[Title/Abstract] OR fear of cost*[Title/Abstract] OR cost of treatment[Title/Abstract])

Concept 1 AND Concept 2

(("Breast Neoplasms"[Mesh]) OR (breast cancer*[Title/Abstract] OR breast neoplasm*[Title/Abstract] OR breast tumour*[Title/Abstract] OR breast tumor*[Title/Abstract] OR breast carcinoma*[Title/Abstract] OR carcinoma of the breast[Title/Abstract] OR carcinoma in situ[Title/Abstract] OR mammary cancer*[Title/Abstract] OR cancer of breast[Title/Abstract] OR cancer of the breast[Title/Abstract])) AND (("Financial Stress"[Mesh] OR "Health Expenditures"[Mesh]) OR (Financial Stress*[Title/Abstract] OR Financial Pressure*[Title/Abstract] OR Financial Hardship*[Title/Abstract] OR Financial Challenges*[Title/Abstract] OR Financial burden*[Title/Abstract] OR health expenditure*[Title/Abstract] OR direct expenditure*[Title/Abstract] OR out-of-pocket expenditure*[Title/Abstract] OR out-of pocket expenditure*[Title/Abstract] OR out of pocket expenditure*[Title/Abstract] OR out-of-pocket expense*[Title/Abstract] OR out-of pocket expense*[Title/Abstract] OR out of pocket expense*[Title/Abstract] OR out-of-pocket cost*[Title/Abstract] OR out-of pocket cost*[Title/Abstract] OR out of pocket cost*[Title/Abstract] OR out-of-pocket payment*[Title/Abstract] OR out-of pocket payment*[Title/Abstract] OR out of pocket payment*[Title/Abstract] OR out-of-pocket spending[Title/Abstract] OR out-of pocket spending[Title/Abstract] OR out of pocket spending[Title/Abstract] OR indirect expenditure*[Title/Abstract] OR expenditure*[Title/Abstract] OR indirect cost*[Title/Abstract] OR fear of cost*[Title/Abstract] OR cost of treatment[Title/Abstract]))

980 results

EMBASE

Concept 1 Breast Cancer
Controlled Vocabulary
'breast cancer'/exp

Synonyms/Keywords
breast cancer* OR breast neoplasm* OR breast tumour* OR breast tumor* OR breast carcinoma* OR carcinoma of the breast OR carcinoma in situ OR mammary cancer* OR cancer of breast OR cancer of the breast

Adjacency Searching
(breast* NEAR/3 (cancer* OR tumor* OR tumour* OR neoplasm* OR adenocarcinoma* OR carcinoma* OR malignan* OR 'in situ')):ti,ab,kw

Syntax

© 2023 Ehsan AN et al. *JAMA Network Open.*

('breast cancer'/exp OR (breast* NEAR/3 (cancer* OR tumor* OR tumour* OR neoplasm* OR adenocarcinoma* OR carcinoma* OR malignan* OR 'in situ')):ti,ab,kw) OR 'breast cancer*':ti,ab,kw OR 'breast neoplasm*':ti,ab,kw OR 'breast tumour*':ti,ab,kw OR 'breast tumor*':ti,ab,kw OR 'breast carcinoma*':ti,ab,kw OR 'carcinoma of the breast':ti,ab,kw OR 'carcinoma in situ':ti,ab,kw OR 'mammary cancer*':ti,ab,kw OR 'cancer of breast':ti,ab,kw OR 'cancer of the breast':ti,ab,kw

Concept 2
Controlled Vocabulary
'financial stress'/exp OR 'financial burden'/exp OR 'out of pocket cost'/exp OR 'out of pocket expenditure'/exp OR 'out of pocket payment'/exp OR 'out of pocket spending'/exp OR 'treatment cost'/exp OR 'financial hardship'/exp OR 'health care cost'/exp

Synonyms/Keywords
Financial Stress* OR Financial Pressure* OR Financial Hardship* OR Financial Challenges* OR Financial burden* OR health expenditure* OR direct expenditure* OR out-of-pocket expenditure* OR out-of pocket expenditure* OR out of pocket expenditure* OR out-of-pocket expense* OR out-of pocket expense* OR out of pocket expense* OR out-of-pocket cost* OR out-of pocket cost* OR out of pocket cost* OR out-of-pocket payment* OR out-of pocket payment* OR out of pocket payment* OR out-of-pocket spending OR out-of pocket spending OR out of pocket spending OR indirect expenditure* OR expenditure* OR indirect cost* OR fear of cost* OR cost of treatment OR capital expenditure* OR direct service cost* OR health expenditure* OR healthcare expenditure* OR health care expenditure*

Syntax
('financial stress'/exp OR 'financial burden'/exp OR 'out of pocket cost'/exp OR 'out of pocket expenditure'/exp OR 'out of pocket payment'/exp OR 'out of pocket spending'/exp OR 'treatment cost'/exp OR 'financial hardship'/exp OR 'health care cost'/exp) OR 'financial stress*':ti,ab,kw OR 'financial pressure*':ti,ab,kw OR 'financial hardship*':ti,ab,kw OR 'financial challenges*':ti,ab,kw OR 'financial burden*':ti,ab,kw OR 'direct expenditure*':ti,ab,kw OR 'out-of-pocket expenditure*':ti,ab,kw OR 'out-of pocket expenditure*':ti,ab,kw OR 'out of pocket expenditure*':ti,ab,kw OR 'out-of-pocket expense*':ti,ab,kw OR 'out-of pocket expense*':ti,ab,kw OR 'out of pocket expense*':ti,ab,kw OR 'out-of-pocket cost* or out-of pocket cost*':ti,ab,kw OR 'out of pocket cost*':ti,ab,kw OR 'out-of-pocket payment*':ti,ab,kw OR 'out-of pocket payment*':ti,ab,kw OR 'out of pocket payment*':ti,ab,kw OR 'out-of-pocket spending':ti,ab,kw OR 'out-of pocket spending':ti,ab,kw OR 'out of pocket spending':ti,ab,kw OR 'indirect expenditure*':ti,ab,kw OR expenditure*:ti,ab,kw OR 'indirect cost*':ti,ab,kw OR 'fear of cost*':ti,ab,kw OR 'cost of treatment':ti,ab,kw OR 'capital expenditure*':ti,ab,kw OR 'direct service cost*':ti,ab,kw OR 'health expenditure*':ti,ab,kw OR 'healthcare expenditure*':ti,ab,kw OR 'health care expenditure*':ti,ab,kw

Concept 1 AND Concept 2

(('breast cancer'/exp OR (breast* NEAR/3 (cancer* OR tumor* OR tumour* OR neoplasm* OR adenocarcinoma* OR carcinoma* OR malignan* OR 'in situ')):ti,ab,kw) OR 'breast

© 2023 Ehsan AN et al. *JAMA Network Open.*

cancer*':ti,ab,kw OR 'breast neoplasm*':ti,ab,kw OR 'breast tumour*':ti,ab,kw OR 'breast tumor*':ti,ab,kw OR 'breast carcinoma*':ti,ab,kw OR 'carcinoma of the breast':ti,ab,kw OR 'carcinoma in situ':ti,ab,kw OR 'mammary cancer*':ti,ab,kw OR 'cancer of breast':ti,ab,kw OR 'cancer of the breast':ti,ab,kw) AND (('financial stress'/exp OR 'financial burden'/exp OR 'out of pocket cost'/exp OR 'out of pocket expenditure'/exp OR 'out of pocket payment'/exp OR 'out of pocket spending'/exp OR 'treatment cost'/exp OR 'financial hardship'/exp OR 'health care cost'/exp) OR 'financial stress*':ti,ab,kw OR 'financial pressure*':ti,ab,kw OR 'financial hardship*':ti,ab,kw OR 'financial challenges*':ti,ab,kw OR 'financial burden*':ti,ab,kw OR 'direct expenditure*':ti,ab,kw OR 'out-of-pocket expenditure*':ti,ab,kw OR 'out-of pocket expenditure*':ti,ab,kw OR 'out of pocket expenditure*':ti,ab,kw OR 'out-of-pocket expense*':ti,ab,kw OR 'out-of pocket expense*':ti,ab,kw OR 'out of pocket expense*':ti,ab,kw OR 'out-of-pocket cost* or out-of pocket cost*':ti,ab,kw OR 'out of pocket cost*':ti,ab,kw OR 'out-of-pocket payment*':ti,ab,kw OR 'out-of pocket payment*':ti,ab,kw OR 'out of pocket payment*':ti,ab,kw OR 'out-of-pocket spending':ti,ab,kw OR 'out-of pocket spending':ti,ab,kw OR 'out of pocket spending':ti,ab,kw OR 'indirect expenditure*':ti,ab,kw OR expenditure*:ti,ab,kw OR 'indirect cost*':ti,ab,kw OR 'fear of cost*':ti,ab,kw OR 'cost of treatment':ti,ab,kw OR 'capital expenditure*':ti,ab,kw OR 'direct service cost*':ti,ab,kw OR 'health expenditure*':ti,ab,kw OR 'healthcare expenditure*':ti,ab,kw OR 'health care expenditure*':ti,ab,kw)

Results 7721

Concept 1 AND Concept 2 WITHOUT health care cost controlled vocabulary term

(('breast cancer'/exp OR (breast* NEAR/3 (cancer* OR tumor* OR tumour* OR neoplasm* OR adenocarcinoma* OR carcinoma* OR malignan* OR 'in situ')):ti,ab,kw) OR 'breast cancer*':ti,ab,kw OR 'breast neoplasm*':ti,ab,kw OR 'breast tumour*':ti,ab,kw OR 'breast tumor*':ti,ab,kw OR 'breast carcinoma*':ti,ab,kw OR 'carcinoma of the breast':ti,ab,kw OR 'carcinoma in situ':ti,ab,kw OR 'mammary cancer*':ti,ab,kw OR 'cancer of breast':ti,ab,kw OR 'cancer of the breast':ti,ab,kw) AND (('financial stress'/exp OR 'financial burden'/exp OR 'out of pocket cost'/exp OR 'out of pocket expenditure'/exp OR 'out of pocket payment'/exp OR 'out of pocket spending'/exp OR 'treatment cost'/exp OR 'financial hardship'/exp) OR 'financial stress*':ti,ab,kw OR 'financial pressure*':ti,ab,kw OR 'financial hardship*':ti,ab,kw OR 'financial challenges*':ti,ab,kw OR 'financial burden*':ti,ab,kw OR 'direct expenditure*':ti,ab,kw OR 'out-of-pocket expenditure*':ti,ab,kw OR 'out-of pocket expenditure*':ti,ab,kw OR 'out of pocket expenditure*':ti,ab,kw OR 'out-of-pocket expense*':ti,ab,kw OR 'out-of pocket expense*':ti,ab,kw OR 'out of pocket expense*':ti,ab,kw OR 'out-of-pocket cost* or out-of pocket cost*':ti,ab,kw OR 'out of pocket cost*':ti,ab,kw OR 'out-of-pocket payment*':ti,ab,kw OR 'out-of pocket payment*':ti,ab,kw OR 'out of pocket payment*':ti,ab,kw OR 'out-of-pocket spending':ti,ab,kw OR 'out-of pocket spending':ti,ab,kw OR 'out of pocket spending':ti,ab,kw OR 'indirect expenditure*':ti,ab,kw OR expenditure*:ti,ab,kw OR 'indirect cost*':ti,ab,kw OR 'fear of cost*':ti,ab,kw OR 'cost of treatment':ti,ab,kw OR 'capital expenditure*':ti,ab,kw OR 'direct service cost*':ti,ab,kw OR 'health expenditure*':ti,ab,kw OR 'healthcare expenditure*':ti,ab,kw OR 'health care expenditure*':ti,ab,kw)

Results 1936

© 2023 Ehsan AN et al. *JAMA Network Open.*

Global Health (EBSCO)

Concept 1
Controlled Vocabulary
DE "breast cancer"

Synonyms/Keywords

Syntax
DE "breast cancer" OR AB (breast cancer* OR breast neoplasm* OR breast tumour* OR breast tumor* OR breast carcinoma* OR carcinoma of the breast OR carcinoma in situ OR mammary cancer* OR cancer of breast OR cancer of the breast) OR TI (breast cancer* OR breast neoplasm* OR breast tumour* OR breast tumor* OR breast carcinoma* OR carcinoma of the breast OR carcinoma in situ OR mammary cancer* OR cancer of breast OR cancer of the breast)
(DE ("breast cancer") OR TI (breast* N3 (cancer* OR tumor* OR tumour* OR neoplasm* OR adenocarcinoma* OR carcinoma* OR malignan* OR "in situ")) OR AB (breast* N3 (cancer* OR tumor* OR tumour* OR neoplasm* OR adenocarcinoma* OR carcinoma* OR malignan* OR "in situ"))) OR AB (breast cancer* OR breast neoplasm* OR breast tumour* OR breast tumor* OR breast carcinoma* OR carcinoma of the breast OR carcinoma in situ OR mammary cancer* OR cancer of breast OR cancer of the breast) OR TI (breast cancer* OR breast neoplasm* OR breast tumour* OR breast tumor* OR breast carcinoma* OR carcinoma of the breast OR carcinoma in situ OR mammary cancer* OR cancer of breast OR cancer of the breast)
Concept 2
Controlled Vocabulary

Synonyms/Keywords

Syntax
AB (Financial Stress* OR Financial Pressure* OR Financial Hardship* OR Financial Challenges* OR Financial burden* OR health expenditure* OR direct expenditure* OR out-of-pocket expenditure* OR out-of pocket expenditure* OR out of pocket expenditure* OR out-of-pocket expense* OR out-of pocket expense* OR out of pocket expense* OR out-of-pocket cost* OR out-of pocket cost* OR out of pocket cost* OR out-of-pocket payment* OR out-of pocket payment* OR out of pocket payment* OR out-of-pocket spending OR out-of pocket spending OR out of pocket spending OR indirect expenditure* OR expenditure* OR indirect cost* OR fear of cost* OR cost of treatment OR capital expenditure* OR direct service cost* OR health expenditure* OR healthcare expenditure* OR health care expenditure*) OR TI (Financial Stress* OR Financial Pressure* OR Financial Hardship* OR Financial Challenges* OR Financial burden* OR health expenditure* OR direct expenditure* OR out-of-pocket expenditure* OR out-of pocket expenditure* OR out of pocket expenditure* OR out-of-pocket expense* OR out-of pocket expense* OR out of pocket expense* OR out-of-pocket cost* OR out-of pocket cost* OR out of pocket cost* OR out-of-pocket payment* OR out-of pocket payment* OR out of pocket payment* OR out-of-pocket spending OR out-of pocket spending

© 2023 Ehsan AN et al. *JAMA Network Open.*

OR out of pocket spending OR indirect expenditure* OR expenditure* OR indirect cost* OR fear of cost* OR cost of treatment OR capital expenditure* OR direct service cost* OR health expenditure* OR healthcare expenditure* OR health care expenditure*)

Concept 1 AND Concept 2
(DE ("breast cancer") OR TI (breast* N3 (cancer* OR tumor* OR tumour* OR neoplasm* OR adenocarcinoma* OR carcinoma* OR malignan* OR "in situ")) OR AB (breast* N3 (cancer* OR tumor* OR tumour* OR neoplasm* OR adenocarcinoma* OR carcinoma* OR malignan* OR "in situ")) OR AB (breast cancer* OR breast neoplasm* OR breast tumour* OR breast tumor* OR breast carcinoma* OR carcinoma of the breast OR carcinoma in situ OR mammary cancer* OR cancer of breast OR cancer of the breast) OR TI (breast cancer* OR breast neoplasm* OR breast tumour* OR breast tumor* OR breast carcinoma* OR carcinoma of the breast OR carcinoma in situ OR mammary cancer* OR cancer of breast OR cancer of the breast)) AND (AB (Financial Stress* OR Financial Pressure* OR Financial Hardship* OR Financial Challenges* OR Financial burden* OR health expenditure* OR direct expenditure* OR out-of-pocket expenditure* OR out-of pocket expenditure* OR out of pocket expenditure* OR out-of-pocket expense* OR out-of pocket expense* OR out of pocket expense* OR out-of-pocket cost* OR out-of pocket cost* OR out of pocket cost* OR out-of-pocket payment* OR out-of pocket payment* OR out of pocket payment* OR out-of-pocket spending OR out-of pocket spending OR out of pocket spending OR indirect expenditure* OR expenditure* OR indirect cost* OR fear of cost* OR cost of treatment OR capital expenditure* OR direct service cost* OR health expenditure* OR healthcare expenditure* OR health care expenditure*) OR TI (Financial Stress* OR Financial Pressure* OR Financial Hardship* OR Financial Challenges* OR Financial burden* OR health expenditure* OR direct expenditure* OR out-of-pocket expenditure* OR out-of pocket expenditure* OR out of pocket expenditure* OR out-of-pocket expense* OR out-of pocket expense* OR out of pocket expense* OR out-of-pocket cost* OR out-of pocket cost* OR out of pocket cost* OR out-of-pocket payment* OR out-of pocket payment* OR out of pocket payment* OR out-of-pocket spending OR out-of pocket spending OR out of pocket spending OR indirect expenditure* OR expenditure* OR indirect cost* OR fear of cost* OR cost of treatment OR capital expenditure* OR direct service cost* OR health expenditure* OR healthcare expenditure* OR health care expenditure*))

287 results

Global Index Medicus
Concept 1
"breast cancer*" OR "breast neoplasm*" OR "breast tumour*" OR "breast tumor*" OR "breast carcinoma*" OR "carcinoma of the breast" OR "carcinoma in situ" OR "mammary cancer*" OR "cancer of breast " OR "cancer of the breast"

Concept 2
"Financial Stress*" OR "Financial Pressure*" OR "Financial Hardship*" OR "Financial Challenges*" OR "Financial burden*" OR "health expenditure*" OR "direct expenditure*" OR "out-of-pocket expenditure*" OR "out-of pocket expenditure*" OR "out of pocket expenditure*" OR "out-of-pocket expense*" OR "out-of pocket expense*" OR "out of pocket expense*" OR "out-of-pocket cost*" OR "out-of pocket cost*" OR "out of pocket cost*" OR "out-of-pocket

© 2023 Ehsan AN et al. *JAMA Network Open.*

payment*" OR "out-of pocket payment*" OR "out of pocket payment*" OR "out-of-pocket spending" OR "out-of pocket spending" OR "out of pocket spending" OR "indirect expenditure*" OR "expenditure*" OR "indirect cost*" OR "fear of cost*" OR "cost of treatment"

("breast cancer*" OR "breast neoplasm*" OR "breast tumour*" OR "breast tumor*" OR "breast carcinoma*" OR "carcinoma of the breast" OR "carcinoma in situ" OR "mammary cancer*" OR "cancer of breast " OR "cancer of the breast") AND ("Financial Stress*" OR "Financial Pressure*" OR "Financial Hardship*" OR "Financial Challenges*" OR "Financial burden*" OR "health expenditure*" OR "direct expenditure*" OR "out-of-pocket expenditure*" OR "out-of pocket expenditure*" OR "out of pocket expenditure*" OR "out-of-pocket expense*" OR "out-of pocket expense*" OR "out of pocket expense*" OR "out-of-pocket cost*" OR "out-of pocket cost*" OR "out of pocket cost*" OR "out-of-pocket payment*" OR "out-of pocket payment*" OR "out of pocket payment*" OR "out-of-pocket spending" OR "out-of pocket spending" OR "out of pocket spending" OR "indirect expenditure*" OR "expenditure*" OR "indirect cost*" OR "fear of cost*" OR "cost of treatment")

7950 results (3080 in English)

("breast cancer*" OR "breast neoplasm*" OR "breast tumour*" OR "breast tumor*" OR "breast carcinoma*" OR "carcinoma of the breast" OR "carcinoma in situ" OR "mammary cancer*" OR "cancer of breast " OR "cancer of the breast") AND ("out-of-pocket expenditure*" OR "out-of pocket expenditure*" OR "out of pocket expenditure*" OR "out-of-pocket expense*" OR "out-of pocket expense*" OR "out of pocket expense*" OR "out-of-pocket cost*" OR "out-of pocket cost*" OR "out of pocket cost*" OR "out-of-pocket payment*" OR "out-of pocket payment*" OR "out of pocket payment*" OR "out-of-pocket spending" OR "out-of pocket spending" OR "out of pocket spending")

("breast cancer*" OR "breast neoplasm*" OR "breast tumour*" OR "breast tumor*" OR "breast carcinoma*" OR "carcinoma of the breast" OR "carcinoma in situ" OR "mammary cancer*" OR "cancer of breast " OR "cancer of the breast") AND ("out-of-pocket")
8 results

("breast cancer*" OR "breast neoplasm*" OR "breast tumour*" OR "breast tumor*" OR "breast carcinoma*" OR "carcinoma of the breast" OR "carcinoma in situ" OR "mammary cancer*" OR "cancer of breast " OR "cancer of the breast") AND ("out-of-pocket" OR "financial stress" OR "health expenditure")
11

© 2023 Ehsan AN et al. *JAMA Network Open.*

eFigure 1. Forest Plot for Financial Toxicity Rate, Subgrouped by Study Quality Ratings Among High-Income Countries (HICs)

Group by Study quality	Study name	Event rate	Lower limit	Upper limit
Fair	Longo CJ, 2011	0.31	0.22	0.42
Fair	Jones SMW, 2018	0.66	0.57	0.74
Fair	Palmer JD, 2018	0.28	0.16	0.44
Fair	Goldberg SL, 2019	0.33	0.26	0.40
Fair	Han S, 2019	0.84	0.77	0.89
Fair		0.50	0.35	0.65
Good	Housser E, 2013	0.10	0.06	0.16
Good	Irwin BB, 2014	0.44	0.36	0.53
Good	Nekhlyudov L, 2016	0.22	0.16	0.30
Good	Knight TG, 2018	0.26	0.23	0.30
Good	Hastart TA, 2019	0.48	0.43	0.53
Good	Perry LM, 2019	0.27	0.20	0.35
Good	Subramanian S, 2020	0.32	0.29	0.35
Good	Wan C, 2021	0.11	0.06	0.18
Good	Offodile AC, 2021	0.51	0.47	0.55
Good		0.29	0.20	0.39
Overall		0.35	0.27	0.44

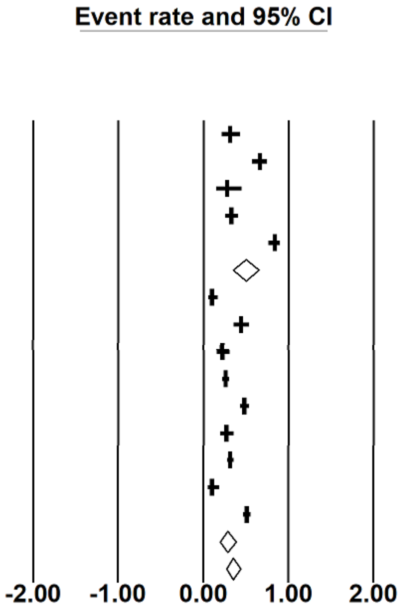

Solid squares represent the rate of financial toxicity reported by each study; horizontal lines denote 95% confidence intervals (CIs). The center of the diamonds represents the pooled rate for financial toxicity for each subgroup and the width represents the respective 95% CI. The size of the solid squares is proportional to the weight of the study. P-value comparing both groups: <0.001.

© 2023 Ehsan AN et al. *JAMA Network Open.*

eFigure 2. Funnel Plot of Standard Error by Logit Event Rate for Studies Reporting High-Income Countries (HICs)

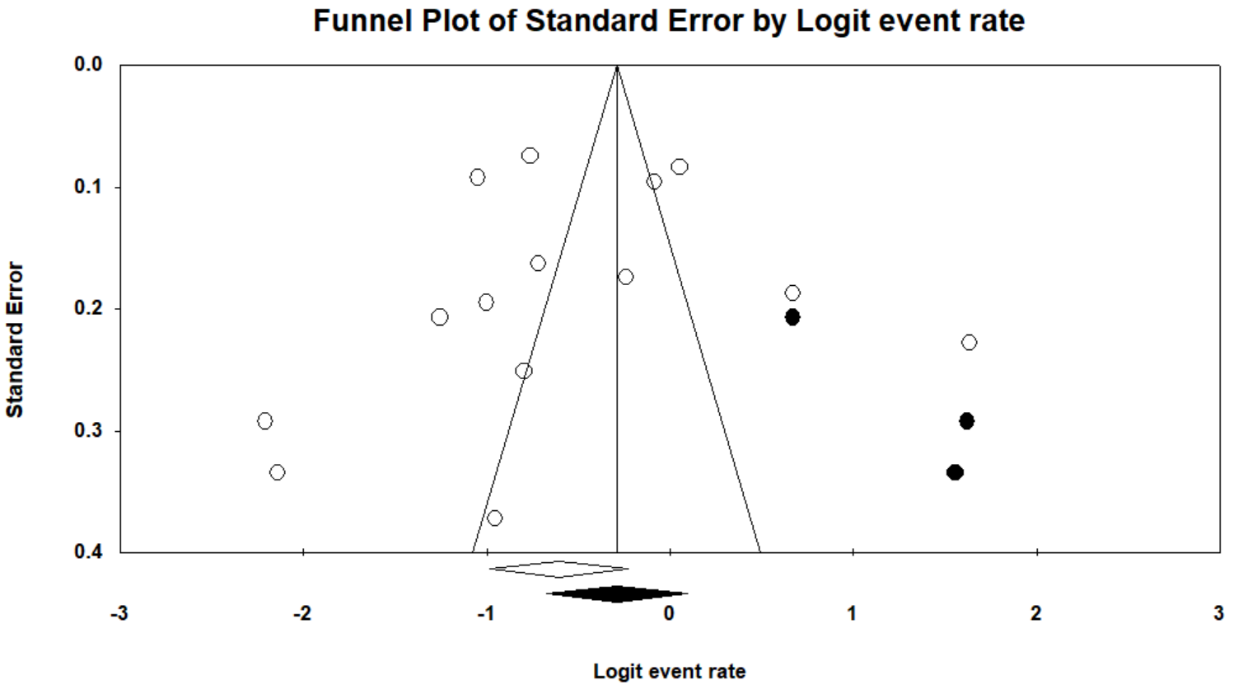

The vertical solid line is drawn at the pooled logit of the financial toxicity (FT) rate, and the other two lines represent the expected 95% confidence interval for a given standard error. The funnel plot visually showed a slight asymmetry towards the right of the pooled point estimate for studies conducted in HICs; however, both Begg's rank correlation test (p=0.62) and Egger's linear regression test (p=0.68) indicated no small study bias.

© 2023 Ehsan AN et al. *JAMA Network Open.*
