# Supplementary material for: Financial Toxicity Among Patients With Breast Cancer Worldwide: A Systematic Review and Meta-analysis
Source: JAMA Netw Open. 2023 Feb 8;6(2):e2255388. doi: 10.1001/jamanetworkopen.2022.55388 (PMC9909501; doi:10.1001/jamanetworkopen.2022.55388)
Supplement: Supplement 2. — Data Sharing Statement [file jamanetwopen-e2255388-s002.pdf]

Data Sharing Statement

Ehsan. Financial Toxicity Among Patients With Breast Cancer Worldwide. *JAMA Netw Open*. Published February 08, 2023. doi:10.1001/jamanetworkopen.2022.55388

Data
Data available: No

Additional Information
Explanation for why data not available: If someone wishes to replicate the study the original dataset is not required the search strategy is, which has been provided in the supplemental online content file. Any extraction of the original dataset has been completed and reported and is unlikely to allow additional statistical analysis.
